# Childcare Subsidy Enrollment Income Generosity and Child Maltreatment

**DOI:** 10.3390/children10010064

**Published:** 2022-12-28

**Authors:** J. Bart Klika, Kathryn Maguire-Jack, Megan Feely, William Schneider, Garrett T. Pace, Whitney Rostad, Catherine A. Murphy, Melissa T. Merrick

**Affiliations:** 1 Prevent Child Abuse America, Chicago, IL 60604, USA; 2 School of Social Work, University of Michigan, Ann Arbor, MI 48116, USA; 3 School of Social Work, University of Connecticut, Hartford, CT 06103, USA; 4 School of Social Work, University of Illinois, Urbana, IL 61801, USA; 5 School of Social Work, University of Nevada, Las Vegas, NV 89154, USA; 6 Abt Associates, Cambridge, MA 02138, USA

**Keywords:** child abuse, child neglect, child maltreatment, childcare subsidies, social welfare policy

## Abstract

In the United States, childcare subsidies are available to low-income working parents to assist with the cost of childcare. The subsidies are provided as block grants to states, which allows for a great deal of flexibility in the specific policies guiding their distribution. Prior research has found a protective link between childcare subsidies and child maltreatment, but the variations in policies have been much less explored. The current study used longitudinal administrative child welfare data from 10 years (2009–2019) linked with state policies regarding the income eligibility requirements of states to examine the impact of these policies on child abuse and neglect among young children (0–5); early school-age children (6–12), and older children (13–17). Using multiple regression and controlling for state demographic characteristics, the study found that more generous policies surrounding income eligibility were related to lower rates of child abuse and neglect investigations at the state level.

## 1. Introduction

Child maltreatment—which encompasses adverse experiences such as neglect and physical abuse—is a significant public health problem. Estimates suggest that by age 18, 37% of children in the United States experience a child protective services investigation [1] and 13% experience a confirmed case of child maltreatment [2]. Child maltreatment hinders children’s behavioral development [3] and leads to substantial societal costs in the immediate and long-term [4]. Given the prevalence of child maltreatment and the negative outcomes associated with it, there is an urgent need to evaluate prevention approaches.

One approach to prevent child maltreatment is to increase economic support for families [5]. Because poverty and economic stress are risk factors for child maltreatment, policies that increase families’ economic security have immense potential for child maltreatment prevention. One such policy is childcare subsidies. Affordable childcare enables caregivers to maintain paid employment while they have young children. Childcare subsidies pay for all or a portion of childcare costs for families with low incomes. In 2018, around 1.3 million children received childcare that was funded by childcare subsidies [6] suggesting this is a significant program that may reduce the risk of child maltreatment for many families.

Although a few studies have examined the relationship between childcare subsidy receipt and child maltreatment in population-based samples of families [7,8], only one study to date [9] has examined the macro-level relationship between childcare subsidy policies and official indicators of child maltreatment. This study leverages macro-level variation built into the policy. Specifically, the federal government funds states’ childcare subsidy programs via a block grant which allows states considerable administrative flexibility in how they establish income eligibility for the program. In other words, some states are more generous than others in who is deemed eligible. In this study, the authors examine the relationship between the relative generosity of states’ income eligibility for childcare subsidies and child maltreatment referral rates.

## 2. Background

### 2.1. Child Maltreatment

Child abuse and neglect pose a significant threat to the long-term wellbeing of children. According to administrative child welfare data, in 2020, nearly 618,000 children were determined, through formal investigation, to be victims of child abuse or neglect [10]. The rate of child victimization was approximately 8.4 cases per 1000 for all children with those under the age of 1 year having the highest rate of victimization at 25.1 cases per 1000 children. The largest portion of cases that come to the attention of child welfare systems are for child neglect (76%), followed by physical abuse (17%), then child sexual abuse (9%). It is estimated that, by their eighteenth birthday, approximately 37% of the US population will have contact with the child welfare system due to concerns regarding abuse or neglect [1] and 13% of children will have a confirmed case of abuse or neglect [2]. 

### 2.2. Prevention through Concrete and Economic Supports

A public health approach to the prevention of child abuse and neglect seeks to address the conditions in which children and families live. Conditions include, but are not limited to, the social policies that provide families with necessary financial and concrete supports during the highest period of risk for child maltreatment. For decades, researchers have linked family economic challenges to child maltreatment [11]. More recently, the Centers for Disease Control and Prevention (CDC) conducted a thorough review of the scientific literature and identified concrete and economic support in times of need as the most promising strategy for the prevention of child abuse and neglect [5]. An ever-growing body of research demonstrates that when states invest in robust policies to reduce economic challenges faced by families, especially for families with young children, child maltreatment is reduced [12]. One policy area that has received increased attention over the past few years as a child maltreatment prevention approach is childcare subsidies.

### 2.3. Childcare as a Critical Parenting Support

Access to high-quality, affordable childcare is a critical support that allows parents to work and alleviates the stress associated with paying for quality childcare. Childcare subsidies have been shown to improve outcomes for adult caregivers. For example, parents who receive childcare subsidies are more likely to work standard hours [13], work full time [14], and earn more [15]. Childcare subsidies can increase household income, both directly by reducing out-of-pocket costs for childcare and indirectly by allowing both parents to have paid employment. Accordingly, subsidies free up income that otherwise would have been spent on childcare, allowing caregivers to invest in other necessities to meet the concrete needs of children and the household. 

When parents do not need to worry about finding and paying for childcare, they have more capacity to attend to children’s social and emotional needs. As such, childcare subsidies have been shown to enhance children’s school readiness [16] and promote positive parenting [17]. These services may even help prevent child maltreatment by promoting prosocial parenting norms and offering respite from the demands of caregiving [18]. In this vein, one study found that a higher percentage of 3- and 4-year-olds attending preschool, both locally and in adjacent neighborhoods, was associated with lower rates of maltreatment investigations and substantiations [19]. Relatedly, in helping parents get access to quality care, when families receive childcare subsidy, they are less likely to be investigated for child maltreatment [8] and are less likely to self-report supervisory neglect in survey studies [7].

### 2.4. Childcare Subsidies

Childcare subsidies are intended to improve the financial wellbeing of low-income families, administered through the Child Care Development Fund (CCDF). Established as a block grant in the 1990s, the CCDF provides states with funding to support adults in securing and maintaining employment, job training, or educational activities that will advance employment prospects. Because childcare subsidies are distributed as a block grant, there is a great degree of state variation in program implementation [20]. Key features of state variation include eligibility requirements, waitlists, redetermination requirements, copayments size, provider requirements, and reimbursement rates. Although this variation has potential to influence the likelihood that families receive benefits and how the benefits matter in their lives, much remains unknown about the ways in which this policy variation relates to outcomes among low-income children and families. 

One recent study examined various enrollment policies related to childcare subsidy and found that counting more benefits as income in determining eligibility was related to higher rates of overall maltreatment and abuse reports, that asset tests were related to higher rates of abuse reports, and that exemptions from copayments for families living in poverty were related to lower overall maltreatment rates [9]. These findings are promising and suggest that more generous childcare subsidy policies may be protective against child maltreatment. The current study differs from this prior study in several key ways, first the current study zeroed in on one specific policy dimension; the level of income allowed for initial eligibility. The prior study examined the overall dollar amount of income eligibility levels related to child maltreatment, whereas the current study conceptualizes this variable differently by examining relative differences between states. For the current study, the level of income allowed for a family of three to become initially eligible for childcare subsidy was considered. This value was then divided by the cost-of-living index for the state. The value was then compared to the average across states for each quarter/year, allowing for an estimate of the enrollment income compared to other states, to account for the large variations in cost of living across the country and the generosity of each state’s policy.

### 2.5. Childcare Subsidies and Child Maltreatment

The Family Adjustment and Adaptation Response (FAAR) theory illustrates the pathway between economic resources and the risk of child maltreatment. Providing safe and consistent care requires resources and the ability to make good decisions. FAAR describes families as existing in an equilibrium between resources and demands; when demands outweigh resources families can tip into chaos that is disproportionate to the initial resource deficit [21]. That is, small resource deficits can become large and lasting problems. To that end, living in a state with more generous childcare subsidies may increase family economic resources. Using a childcare voucher reduces or eliminates childcare expenses, which increases a family’s net income, allows resources to meet household needs in other ways, decreases work disruptions [22], increases stable employment opportunities, and/or simply provides increased stability and access to childcare. At the same time, more generous childcare subsidies may also help to decrease cognitive load, allowing parents to make improved parenting decisions. It is possible that the pathways leading from childcare subsidies to the prevention of child neglect and child physical abuse may follow slightly different paths.

### 2.6. Childcare Subsidies and Neglect

Most maltreatment, 75%, involves neglect, which Feely and colleagues (2020) consider as a gap in safe and consistent care (SCC) and is likely strongly influenced by the resources available to families, rather than intentional acts of harm [23]. Long assumed to be more benign than abuse, recent studies have shown that neglect can be as damaging, and even as fatal, for children as abuse. Strikingly, the etiologies of child abuse and child neglect have long been assumed to be the same, rooted in parental psychopathology, parental knowledge of child development, and bi-directional pathways between children and parents [23,24]. However, it may be that important aspects of child neglect are closely tied to experiences of economic instability [25,26]. Child neglect encompasses a range of parenting lapses including supervisory neglect, or the risk of harm from leaving children unsupervised or with inadequate supervision; exposure neglect, or the risk from exposure to unsafe settings; physical neglect, or the risk of harm from inadequate provision of basic necessities; and medical and educational neglect, or the failure to provide necessary medical or educational access to children. 

Executive functioning, the ability to manage complex tasks, can be impaired by high cognitive load and a scarcity mindset, which affect decision making and are common among people living with chronic financial stress. Cognitive load refers to the amount of information that the working memory can hold [27]. As the amount of information that needs to be maintained increases, cognitive load increases and compromises decision making [28]. Parents who face economic hardship are likely to experience a high cognitive load and a scarcity mindset because they are often managing a host of factors to stretch limited resources in an effort to provide SCC. In this sense, increasing resources should decrease the amount of information that parents need to maintain and/or allow them to do tasks such as making more consistent childcare plans so they are not constantly adding to their cognitive load to reconfigure plans.

Exo-system factors such as social policies have important implications for individual-level experiences of economic hardship and poverty. Access to cash, in-kind, and other instrumental supports may buffer many of the financial-based stressors that are thought to influence the risk of child neglect. For example, economic hardship may result in parents being unable to provide safe and secure housing for children, and child welfare-involved families have high rates of housing instability [29]. Similarly, approximately one in five children are food insecure [30], a primary concern for physical neglect. 

Both supervisory and exposure neglect may also be closely tied to economic hardship. Together, leaving a child alone without a caretaker and leaving a child with an unsuitable caretaker make up approximately 44% of supervisory neglect cases [31]. This may also be linked to economic hardship [32] through parents’ inability to afford childcare, the need to work unstable or variable shift work, or through the necessity of leaving younger children in the care of older children [7]. Recent work by Maguire-Jack and colleagues (2019) indicates that receipt of childcare subsidies is associated with decreased supervisory neglect, likely a result of increased access to safe and stable childcare [7]. 

In sum, economic hardship may result in parents’ inability to provide necessary essentials such as safe housing, safe and consistent childcare, medical care, and nutrition. It may also increase cognitive load in parents, making it more likely that decision making around safety may be impaired. In this sense, living in a state with more generous access to childcare subsidies may reduce the risk of child neglect, particularly supervisory neglect. Additionally, if access to childcare subsidies increases a families’ available cash to spend on other goods it may also decrease the risk of a range of other forms of neglect. 

### 2.7. Childcare Subsidies and Abuse

The vast majority of research indicates that child abuse is closely linked to parental psychopathology [33] and physically and emotionally harsh parenting practices [34]. However, economic hardship may also be linked to an increased likelihood of abuse through the heightened stress experienced by families. Economic Stress Theory [35], suggests that when families experience economic shocks and stressors, maladaptive parenting practices may also increase through a rise in overall stress in the family. The theory specifically suggests that economic hardships elevate the risk of tension and fighting between parents, which then increases the risk of harsh parenting practices [35]. To the extent that childcare subsidies provide economic security and reduce economic shocks to the family, harsh parenting and child abuse may be reduced.

### 2.8. Variations in Childcare Subsidy Policies

Despite research findings that receipt of childcare subsidy may improve parental economic indicators and have preventive benefits against child abuse and neglect, little is known about the ways in which the policy variations across states might differentially relate to abuse and neglect. Prior research suggests that variations in subsidy receipt may have important implications. For example, research in Oregon has shown that longer eligibility periods are associated with less frequent exits from the childcare subsidy program [36]. Similarly, experimental work from Cook County, Illinois has shown that extending the eligibility period of subsidies increases subsidy use. However, that experiment focused on families whose incomes exceeded the state eligibility limits and may not be generalizable to lower-income families [37]. Further, state CCDF implementation flexibility has been found to impact families involved with child protective services. For example, states that are most accommodating in terms of eligibility, priority lists, copays, and activity requirements for child welfare-involved families have significantly fewer children removed from home than less-accommodating states [38].

**Figure 1:**
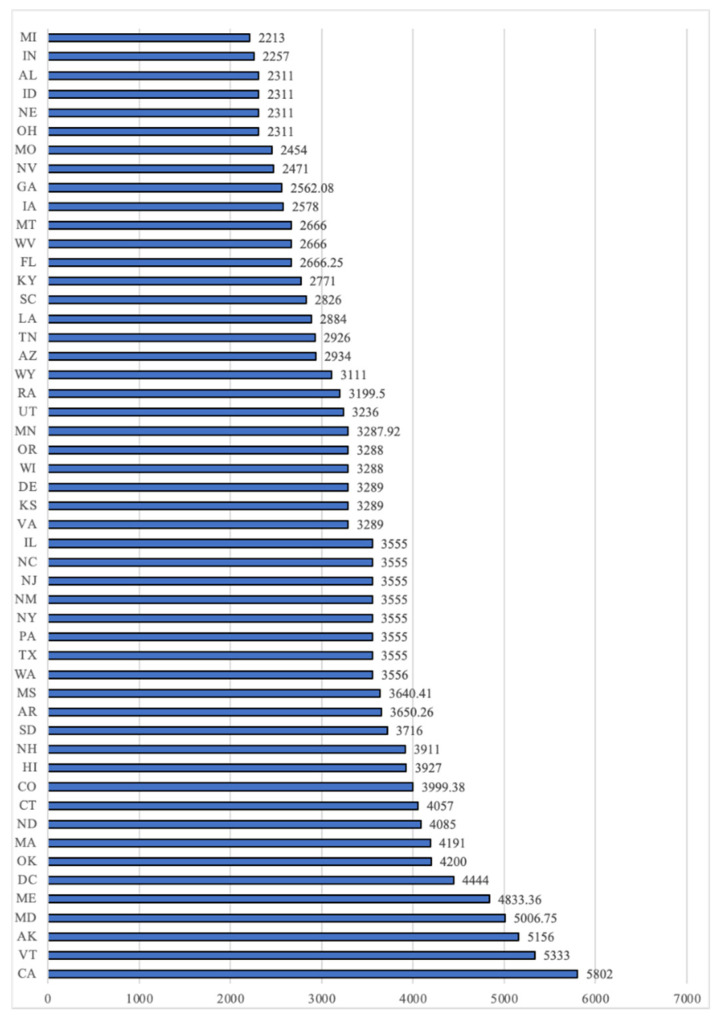
Maximum Monthly Enrollment Income for a Family of Three, 2019. Source of data: Urban Institute Childcare Development Fund Policy Database (ccdf.urban.org accessed 1 January 2022).

Enrollment income refers to the maximum amount of income a family is allowed to earn in order to still qualify to receive the childcare subsidy. Enrollment income varies significantly between states, meaning that a family may qualify for the subsidy in one state but not in another. In 2019, for a family of three, the maximum gross (pre-tax) income a family could earn in a month and still qualify for childcare subsidy ranged from a low of USD2213 in Michigan to USD5802 in California; with an average of USD3408. Figure 1 displays the maximum monthly enrollment income for a family of three in 2019 by state.

### 2.9. Current Study

The current study expands on prior work by Rochford and colleagues [9] in several key ways. First, the current study includes the cost-of-living index to account for the variation in childcare costs across states. Second, the current study includes a different measure of enrollment income, that examines the relative generosity of the policy dimension. Third, the current study examines a longer period (2009 to 2019) compared to the prior study, which examined 2012–2018. The current study examines the relationship between states’ relative childcare subsidy enrollment income threshold and states’ rates of child maltreatment investigations from 2009 to 2019. We examine this relationship by type of maltreatment referral (physical abuse only and neglect only) and by child age (birth to age five, age six to age twelve, and age thirteen to age seventeen). We hypothesize that higher relative generosity in childcare subsidy enrollment income will be negatively correlated with child abuse and neglect investigations among the youngest group of children. The youngest age group is the primary population that childcare subsidies target because most of these children are not yet in school. Childcare subsidies can help alleviate economic hardship and reduce poverty by making paid employment more feasible for caregivers.

## 3. Methods

### 3.1. Data 

Childcare subsidy policy information was obtained from the Urban Institute policy database (ccdf.urban.org accessed 1 January 2022), which provides annual information about state-level variation in policies and procedures regarding CCDF policies. These data were compiled into a state-by-quarter database and linked with child maltreatment data from the National Child Abuse and Neglect Data System (NCANDS), with information on child abuse and neglect investigations by state from 2009 to 2019. We linked in control variables from the United States Census Bureau and American Community Survey (ACS), including population by age and race, urbanicity, poverty rate, and unemployment rate. Finally, we linked in cost-of-living information from The Council for Community and Economic Research (C2ER). C2ER provides quarterly estimates of the relative cost of living for various cities across the United States to provide a cost-of-living index (COLI). For the quarters of study, we averaged the COLI for all available cities within each state to obtain a state COLI. Children were grouped into three age groups: (1) young children, birth age to age 5, when children are expected to benefit the most from childcare subsidies; (2) young school age, age 6–12 when children are within the age group for which parents are still eligible to collect subsidy for before- and after-school care, but it is expected to be less helpful compared to younger age children; and (3) older children that are generally not eligible for the benefit unless they have a disability.

### 3.2. Measures

Key dependent variable. Child maltreatment rates were examined as investigations for all types of maltreatment grouped together, then by physical abuse and neglect separately. The authors examined state rates of child maltreatment investigations among young children, early school-age children, and older children; physical abuse investigations among these three groups, and finally neglect investigations among these three groups. 

Key independent variable. Enrollment income was examined as the maximum monthly income allowed for a family of three divided by the COLI. The average enrollment income divided by COLI was then calculated by quarter year for the United States. The number of standard deviations from the mean was calculated to determine the relative generosity of the enrollment income level for that state in that quarter, with higher values indicating greater generosity.

Control variables. State-level control variables were linked from the US Census Bureau, ACS, one-year estimates for the period 2009–2019. Control variables were selected that have been found in previous research to be related to child maltreatment [39], including racial composition (proportion white, proportion black, proportion some other racial group), proportion of the state considered urban, the unemployment rate, and the poverty rate (percent of resident under the federal poverty level).

### 3.3. Analysis

A series of multiple regressions were run to examine the relationship between state enrollment income generosity and child maltreatment investigations. State and time fixed effects to control for differences across states and time in observable and unobservable predictors were included. State fixed effects adjust for time-invariant factors affecting each state that may be correlated with childcare subsidy and child maltreatment whereas quarterly fixed effects adjust for time shocks which are unobservable that are correlated with childcare subsidy and child maltreatment. The inclusion of both of these factors allows for the isolation of impact of the policy variable of interest. 

The analyses take the form of the general equation:

$${\mathrm{CM}}_{sq}=\;\beta_{0}+\beta_{1}\;CC\;Subsidy\;Income\;Threshold+X_{sq}+\theta_{sq}+\delta_{sq}+\epsilon_{sq}$$

where CM_*sq*_ is the child abuse or neglect investigation rate for that state/quarter, $\beta_{0}$ is the intercept, $\beta_{1}\;CC\;Subsidy\;Income\;Threshold$ is the measure of income threshold generosity, $X_{sq}$ is the state-level covariates, $\theta_{sq}$ is the state fixed effects, $\delta_{sq}$ is the quarterly fixed effects, and $\epsilon_{sq}$ is the error term.

The cluster command was utilized to account for grouping at the state level and produce robust standard errors. A series of six models were estimated: two models (neglect investigations, physical abuse investigations) on each of the three age groups: young children (birth to age five; age six to age twelve; age thirteen to age seventeen). Within each model, the weight command to weight each model on the child population of that specific age group was used, such that, for example, regressions examining investigations among children from birth to age five were weighted on the child population for that state for the same age group. 

## 4. Results

### 4.1. Descriptive Statistics

Table 1 displays the descriptive statistics of the sample. Across the 44 quarters of the 2009–2019 time period, the neglect referral rate was 1149.95 per 100,000 and for physical abuse it was 334.25 per 100,000. Among children aged 6 to 12 years the referral rates were 851.55 per 100,000 for neglect and 337.35 per 100,000 for physical abuse. Among children aged 13 to 17 years, the rates were 549.34 per 100,000 for neglect and 253.89 per 100,000 for physical abuse. The average maximum monthly income to be eligible to receive childcare subsidy for a family of three at the time of enrollment was USD2991, and this ranged from USD838 to USD5802. In terms of control variables, the average percentage of the state population that was white was about 61%, black was about 12%, and other race was about 11%. The average percentage of the state population that was urban was 74%, and the average unemployment rate and poverty rates were 6% and 13%, respectively.

### 4.2. Inferential Statistics

Table 2 shows the results from our regression models. Having a more generous enrollment income was associated with a lower rate of neglect investigations and physical abuse investigations among children from birth to age five. Specifically, one standard deviation increase in generosity of enrollment income is associated with a 3% decrease in neglect investigations and a 4% decrease in physical abuse investigations. These findings were not statistically significant for the early school-age and older children; however, the direction of the coefficients is also negative and of similar magnitude.

## 5. Discussion

Child maltreatment is a costly public health problem that has substantial adverse consequences for children and few effective population-level prevention approaches. This study leveraged state variation in childcare subsidy enrollment income generosity to examine the relationship between childcare subsidies and child maltreatment. The findings identify a scalable and effective maltreatment prevention program for lower-income working families with young children. Expanding the eligibility of childcare subsidy programs by increasing enrollment generosity is associated with lower maltreatment rates. There are a variety of potential pathways through which expanded access to childcare subsidies may be linked to decreased rates of child maltreatment. It may be, for example, that expanding the eligible population of families induces those who are “over” the benefit cliffs of some other social safety net programs to enroll, providing substantial and much-needed support. The association between expanded eligibility and decreased child maltreatment may be due to a multitude of factors across ecological levels [40]. These factors include: decreased parental stress, cognitive load, material hardship, increased access to work, and children’s behavior being positively impacted by high-quality early childcare settings.

States with more generous childcare subsidy income eligibility had lower child physical abuse and neglect referral rates among children from birth to age five, supporting the study hypothesis. Interestingly, although the estimates were not statistically significant among older children, they trended in the same direction of more generous eligibility reducing maltreatment. Considering that a substantially smaller percentage of children in these age groups are cared for through the childcare subsidy, this is potentially an encouraging finding indicating that with wider adoption the effects on reducing maltreatment could be even greater. These results suggest that if states increased the enrollment generosity, i.e., raised the ceiling on the maximum amount of money a family can earn and still qualify for the subsidy, the rates of child maltreatment for children under age five would go down. For example, if in 2019, Michigan increased their enrollment generosity from USD2213 to USD2912, or one standard deviation above the current level, we estimate that 1220 fewer children would be neglected, and 528 fewer children aged five and younger would be physically abused over a one-year period. Such an increase would cap the annual eligible income for a parent and two children at an annual gross income of USD34,944—barely more than 160% of the federal poverty level in the United States. 

States have considerable discretion in setting income thresholds for family support programs. For example, in 2019 maximum earned income for a parent with two children in 2019 who would be eligible for the federal EITC was USD46,703 [41]. This increase is also allowable without changes to current federal policy. The federal government allows states to set income eligibility to as high as 85% of state median income; yet, only Alaska, Arkansas, California, and Maine have done so [42]. Increasing childcare subsidies would bring the enrollment income generosity more in alignment with that support policy for working parents. Although one standard deviation is a modest increase in the expansion of eligibility, these findings suggest that it could result in measurable results for young children. 

Contrary to our expectation [43], the results did not demonstrate a stronger association with neglect than abuse. For the youngest children, the coefficient for abuse was larger than that for neglect and for the older child groups, although not statistically significant, the coefficients were similar. Results from prior studies show a stronger effect on neglect when income increases and in some cases no effect on abuse [44], and the findings on the reduced rates of abuse point toward a number of potential mechanisms linking economic hardship and maltreatment.

In studies where only parent income is affected, but the time and energy demand of caring for their children likely remains relatively constant, parents may be able to provide better physical care, but parental stress and strain likely remain undiminished. However, childcare subsidies increase family financial resources and provide respite for parents from the constant care of children. This pathway likely results in reduced rates of neglect. Similarly, by reducing the financial burdens and the stress of childcare, parents may be better able to use more positive parenting strategies, leading to fewer incidents of escalation of corporal punishment into physical abuse [45].

These findings are in accordance with the Centers for Disease Control (CDC)’s promising strategies for maltreatment prevention by strengthening economic supports for families [46]. Research shows that in addition to economic insufficiency or hardship, economic instability is also a risk factor for maltreatment [47]. This suggests that many families may need more income but also a steadier source of income. Policies that increase family income, such as more generous state Earned Income Tax Credits or a higher minimum wage, lead to a reduction in parent-reported maltreating behaviors and in state-level child neglect rates [29,44,48]. Neither the EITC nor minimum wage is focused on improving parents’ ability to provide SCC, yet the additional income has specific positive externalities for the population most sensitive to changes in these policies. Childcare subsidies may function in a similar way and are similarly scalable, not requiring extensive government investment in implementing other maltreatment prevention programs, such as home visiting. 

For employed parents, childcare is often a large household expense and, in many places, the average cost of having two children in childcare is more expensive than housing [49]. The financial burden of childcare is particularly difficult for parents living at or near the poverty line. In this study, the average income limit for subsidy eligibility was USD2991 per month or a gross annual income of less than USD36,000. This is equivalent to about 170% of the federal poverty line (in 2019, for the same sized family). However, there is a significant gap between when eligibility for subsidies ends and the income level where families can afford childcare along with other basic necessities. In 2019, Mississippi parents at 200% of the federal poverty level paid a lower percentage of their income for childcare than in any other place in the country [49]. Yet, home-based childcare for one child still amounted to 10% of their income and center-based care (the more expensive option) amounted to 14% of their income. The unaffordability of childcare for families who are well above the federal poverty level identifies the significant gap between most states’ current enrollment generosity and the cost of childcare for working families. Expanding the enrollment generosity likely works partially by narrowing the gap between the income at which families qualify for the subsidy and the income level at which they can afford market-rate childcare. Put another way, it extends the benefit cliff of the childcare subsidy closer to the far bank so fewer families fall into the chasm. 

Reducing maltreatment is a positive externality of the childcare subsidy program, that is, the reduction is an additional and unintended benefit of a work-support program. Therefore, in addition to the direct impact on families who benefit from the childcare subsidy, expanding the subsidy reduces a state’s child welfare expenditures and the long-term burden of child maltreatment. The financial gains of this positive externality should be included when the costs of the childcare subsidy are estimated in order to produce an accurate accounting of the expenses and benefits of the program [23]. 

Although the applicability of these results during the height of the pandemic is uncertain, the importance of childcare for individual families and the economy remains undiminished. The data in this paper come from the decade preceding the COVID-19 pandemic and the childcare landscape was reshaped by that global event. During what could be characterized as the initial phase of pandemic-related closures, starting in March 2020, approximately half of childcare facilities closed at least temporarily [50]. Many had reopened by September 2020, but 88% reported reduced capacity [50]. Although some industries have rebounded to pre-pandemic levels, as of July 2022 only 76% of pre-pandemic childcare jobs had been recovered [51]. The simultaneous loss of capacity and high unemployment may have resulted in a vicious cycle of parents who cannot afford childcare without the subsidy but cannot commit to employment without childcare leading to a suppressed demand for childcare options for lower-income families. Increasing income generosity may help support the ongoing recovery of this critical industry.

The results of this study suggest that simple changes to childcare subsidy policies may be a scalable and feasible change that will have significant and relevant outcomes for children and families. Using modifications to existing policies is an efficient approach to strengthening economic support for families and helping them provide safe and consistent care for children.

## 6. Limitations

There are several limitations that must be considered when interpreting the findings of this research. First, the study does not examine the receipt of childcare subsidy at the individual level. Therefore, it does not measure actual childcare subsidy receipt. Instead, it examines the associations between policy variations (level of income allowable for enrollment in childcare subsidy) and child maltreatment rates at the state level. In this way, the study examines the policy impact of choices made during program implementation rather than the impact of an individual parent receiving subsidy. However, such findings have direct relevance for those making policy decisions at the state level. Second, the study does not account for childcare availability and quality. The inclusion of such measures would enhance the study because availability of childcare and the quality thereof vary widely, especially between rural and urban areas. Third, the study relies upon officially reported child maltreatment. Administrative data on child maltreatment include only acts that have been noticed by someone who reports their concerns to child protection authorities. Family violence is often a private matter that many individuals are unwilling to intervene in; therefore, it is commonly acknowledged that the actual occurrence of child maltreatment likely surpasses official maltreatment rates. Further, to the extent that there is bias in who gets reported for child maltreatment, the estimates will also be biased. Fourth, although the study accounts for clustering within states, the study does not account for the clustering of children within individual families. Fifth, although the study included state fixed effects in an attempt to control for other state-level factors that may contribute to the findings and confound the relationship between childcare subsidy policies and child maltreatment investigations, there are local variations in policies within states, for which state fixed effects are unable to control. Sixth, although the current study controlled for poverty rate and racial composition of states, the study did not explicitly examine the ways in which childcare subsidy policies might reduce racial and socioeconomic inequalities in child maltreatment reports. Future studies should investigate these research questions explicitly. Finally, no single policy (or program) will be successful in preventing all cases of child abuse and neglect. Instead, successful child maltreatment prevention requires a robust set of policies and strategies, across the social ecology and across the developmental continuum. In this paper, we looked at a single component of variation in a single policy.

## 7. Conclusions

Children require safe, stable, and nurturing relationships to develop into happy, healthy, and productive adults. Challenging life circumstances, including economic hardship, can overload a parent’s capacity to provide safe and nurturing care to their children. States that provide generous income eligibility for childcare subsidies have the potential to decrease child physical abuse and neglect.

## Figures and Tables

**Table 1:** Descriptive Statistics, N = 2192 state/quarters.

	Mean	Standard Deviation	Range
Young neglect referral rate per 100,000	1149.95	659.75	2.29–4482.19
Young physical abuse referral rate per 100,000	334.25	367.25	1.27–3702.28
Early school-age neglect referral rate per 100,000	851.55	529.76	1.59–3418.53
Early schoolage physical abuse referral rate per 100,000	337.35	269.93	1.83–2818.53
Older child neglect referral rate per 100,000	549.34	368.82	1.22–2291.22
Older child physical abuse referral rate per 100,000	253.89	174.56	1.22–1770.79
Maximum monthly income for family of 3 at enrollment	USD2991	USD700	USD838–USD5802
Proportion of child population white	60.78	17.18	13.04–91.42
Proportion of child population black	11.84	10.55	<1–43.59
Percent of child population race other than white or black	10.83	9.92	3.17–70.38
Proportion of population urban	73.39	14.48	38.2–95
Unemployment rate	6.01	2.33	2.4–13.7
Poverty rate	12.97	3.47	3.7–23.1

**Table 2:** OLS Regression Enrollment Income Adjusted for Cost of Living and Child Maltreatment Investigations (Logged) by Maltreatment Type and Child Age Group 2009–2019 with robust standard errors and clustering at state level N = 2192 state/quarters.

	Neglect Investigations	Physical Abuse Investigations
	Beta (Standard Error)	Beta (Standard Error)
Young children: 0–5		
Enrollment income/cost-of-living index	−0.028 (0.013) *	−0.037 (0.017) *
Proportion children white	−1.084 (6.547)	−5.757 (7.376)
Proportion children black	4.881 (6.698)	−7.120 (11.187)
Proportion children another race	−1.805 (7.804)	−11.750 (11.582)
Proportion population urban	0.029 (0.028)	0.037 (0.027)
Unemployment rate	0.199 (0.296)	−0.038 (0.043)
Poverty rate	0.001 (0.012)	0.006 (0.019)
Early school-age children: 6–12		
Enrollment income/cost-of-living index	−0.031 (0.017) +	−0.033 (0.019) +
Proportion children white	−2.665 (6.888)	−2.598 (5.122)
Proportion children black	2.062 (6.973)	−2.499 (8.445)
Proportion children another race	−2.194 (8.075)	−8.393 (8.286)
Proportion population urban	0.031 (0.038)	0.039 (0.024)
Unemployment rate	0.018 (0.033)	−0.019 (0.034)
Poverty rate	−0.001 (0.014)	0.004 (0.015)
Older children: 13–17		
Enrollment income/cost-of-living index	−0.031 (0.017) +	−0.037 (0.015) +
Proportion children white	1.812 (5.906)	4.300 (4.031)
Proportion children black	7.691 (6.826)	4.261 (6.905)
Proportion children another race	−2.281 (8.588)	−7.355 (8.134)
Proportion population urban	0.030 (0.038)	0.034 (0.019) +
Unemployment rate	0.036 (0.030)	0.010 (0.024)
Poverty rate	−0.002 (0.013)	−0.001 (0.012)

* *p* < 0.05; + *p* < 0.10; State and quarterly fixed effects not shown. Weighted for child population of each specific age group.

## Data Availability

The analyses presented in this publication were based on data from National Child Abuse and Neglect Data System (NCANDS). These data were provided by the National Data Archive on Child Abuse and Neglect at Cornell University and Duke University and have been used with permission. The data were originally collected under the auspices of Prevent Child Abuse America. Funding was provided by Centers for Disease Control and Prevention. The collector of the original data, the funder, NDACAN, Cornell University, and the agents or employees of these institutions bear no responsibility for the analyses or interpretations presented here. The information and opinions expressed reflect solely the opinions of the authors.
